# Small extracellular vesicles derived from dermal fibroblasts promote fibroblast activity and skin development through carrying miR-218 and ITGBL1

**DOI:** 10.1186/s12951-022-01499-2

**Published:** 2022-06-22

**Authors:** Qin Zou, Mei Zhang, Rong Yuan, Yifei Wang, Zhengyin Gong, Rui Shi, Yujing Li, Kaixin Fei, Chenggang Luo, Ying Xiong, Ting Zheng, Li Zhu, Guoqing Tang, Mingzhou Li, Xuewei Li, Yanzhi Jiang

**Affiliations:** 1 Department of Zoology, College of Life Science, Sichuan Agricultural University, Ya’an, 625014 Sichuan China; 2 Chengdu Livestock and Poultry Genetic Resources Protection Center, Chengdu, 610081 Sichuan China; 3 Institute of Animal Genetics and Breeding, College of Animal Science and Technology, Sichuan Agricultural University, Chengdu, 611130 Sichuan China

**Keywords:** Dermal fibroblasts, Small extracellular vesicles, Fibroblast activity, Skin development, miR-218, ITGBL1

## Abstract

Skin thickness is closely related to the appearance of human skin, such as sagging and wrinkling, which primarily depends on the level of collagen I synthesized by dermal fibroblasts (DFs). Small extracellular vesicles (SEVs), especially those derived from human DFs (HDFs), are crucial orchestrators in shaping physiological and pathological development of skin. However, the limited supply of human skin prevents the production of a large amount of HDFs-SEVs, and pig skin is used as a model of human skin. In this study, SEVs derived from DFs of Chenghua pigs (CH-SEVs), considered to have superior skin thickness, and Large White pigs (LW-SEVs) were collected to compare their effects on DFs and skin tissue. Our results showed that, compared with LW-SEVs, CH-SEVs more effectively promoted fibroblast proliferation, migration, collagen synthesis and contraction; in addition, in mouse model injected with both SEVs, compared with LW-SEVs, CH-SEVs increased the skin thickness and collagen I content more effectively. Some differentially expressed miRNAs and proteins were found between CH-SEVs and LW-SEVs by small RNA-seq and LC–MS/MS analysis. Interestingly, we identified that CH-SEVs were enriched in miRNA-218 and ITGBL1 protein, which played important roles in promoting fibroblast activity via activation of the downstream TGFβ1-SMAD2/3 pathway in vitro. Furthermore, overexpression of miRNA-218 and ITGBL1 protein increased the thickness and collagen I content of mouse skin in vivo. These results indicate that CH-SEVs can effectively stimulate fibroblast activity and promote skin development and thus have the potential to protect against and repair skin damage.

**Graphical Abstract:**

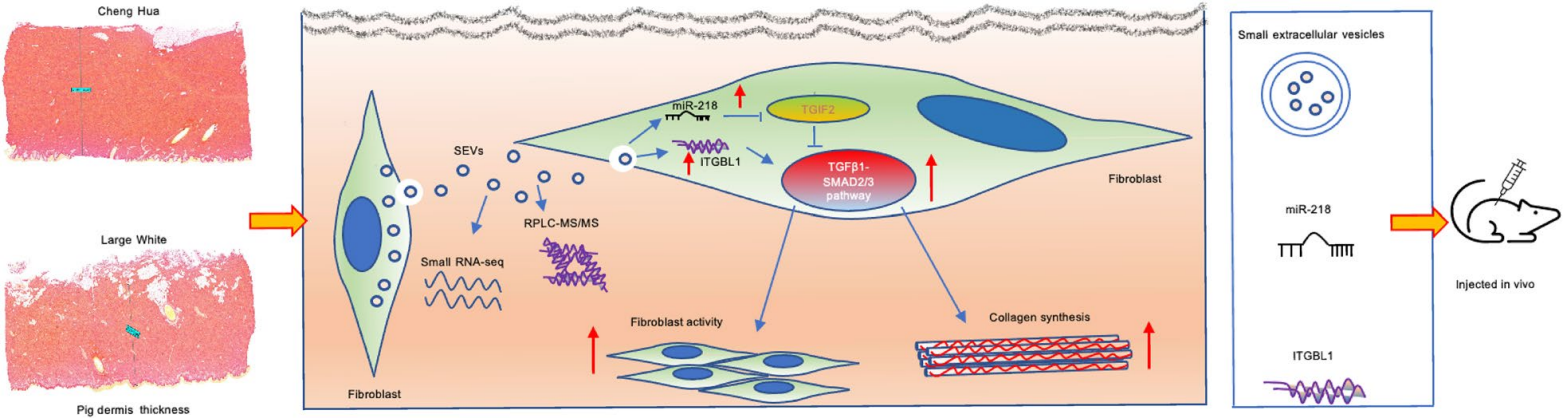

**Supplementary Information:**

The online version contains supplementary material available at 10.1186/s12951-022-01499-2.

## Introduction

Skin thickness is closely related to the appearance of human skin, such as sagging and wrinkling, which primarily depends on the level of collagen I synthesized by fibroblasts in the dermal layer [1]. Human skin thickness varies considerably according to race, age, sex, and region of the body surface [2, 3]. As life progresses, physical changes in the skin are the first observation demonstrating organismal ageing, and the skin thickness, number and biosynthetic capacity of fibroblasts, and collagen content decreases with age [4–6], which may account, in part, for the wrinkling and loss of elasticity [7]. Although various materials, such as antioxidants, retinoids, peptides, growth factors, and dermal fillers have been used to protect or repair the skin [8–11], most of the available skin treatment products lack the capacity to enrich the skin completely.

Small extracellular vesicles (SEVs), generally called exosomes, are cell-derived nanoscale vesicles with a diameter of 40–160 nm that can mediate cell-to-cell communication and regulate the properties of target cells through factors such as proteins, nucleic acids, carbohydrates, and lipids [12]. Previous reports demonstrated that multiple kinds of endogenous SEVs, such as those derived from adipose-derived stem cells (ADSCs) and mesenchymal stem cells (MSCs), are crucial orchestrators in shaping the physiological and pathological development of the skin [13–16]. Moreover, recent studies have discovered that SEVs derived from autologous human dermal fibroblasts (HDFs) can more efficiently ameliorate skin ageing [17, 18] and promote cutaneous wound healing [19, 20], which indicates that HDF-SEVs may be potential materials for protecting against and repairing skin damage.

However, because human skin is obtained during plastic surgery procedures or from deceased donors, the limited supply of human skin severely prevents the production of a large amount of HDF-SEVs. Porcine skin has been used as a model of human skin because it shares histological, ultrastructural, and physiological skin attributes with those of human skin [21, 22]. The Chenghua pig (CH) is an endangered black breed native to southwestern China in Sichuan Province and is characterized by a superior skin thickness of approximately 8 mm or more at the back in adult individuals [23]; however, the most popular commercial pig breeds, such as Large White pigs (LW) and Landrace pigs, only show a thickness of approximately 2 mm at the back in adult individuals [21, 24]. These studies indicate that CH pigs may have strong DF activity and skin development and may be an ideal model animal for researching mammalian skin biology.

Here, we hypothesized that SEVs derived from CHDFs could have a beneficial effect on fibroblast activity and skin development via the enriched alterations of proteins and miRNA cargos. To test this hypothesis, we isolated SEVs derived from CHDFs and LWDFs and comparatively investigated their effects on fibroblast activity in vitro and skin development in vivo. Moreover, we identified CH-SEVs enriched in miRNA-218 and the ITGBL1 protein and explored their molecular mechanism in regulating fibroblast activity in vitro and their roles in skin development in vivo.

## Result

### Characterization of SEVs derived from CHDFs and LWDFs

**Fig. 1:**
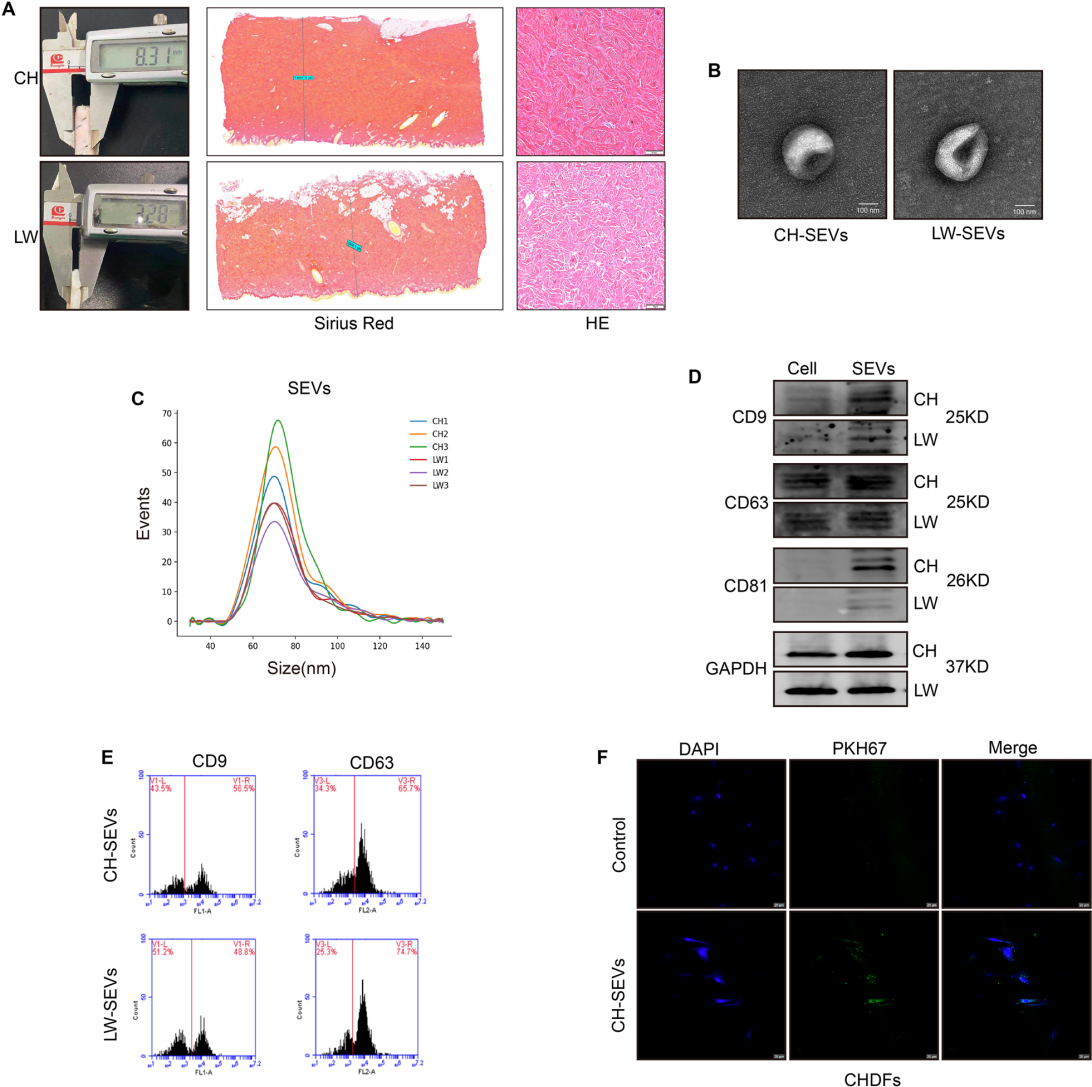
Characterization of skin thickness and SEVs from DFs. **A** Skin thickness (left), Sirius red (middle), and HE stain (right) of CH and LW pig skin tissue. Scare bars of Sirius/HE = 1000/100 µm. **B** Transmission electron microscope images of SEVs derived from DFs of CH and LW pigs. Scare bars = 100 nm. **C** Size distribution of CH/LW-SEVs by nanoparticle tracking analysis. n = 3. **D** Western blot bands of CD9, CD63, and CD81 in secreting cells and CH/LW-SEVs. n = 3. **E** CD9 and CD63 of CH/LW-SEVs were detected by flow cytometry. n = 3. **F** Images of location between CH-SEVs and CHDFs by confocal microscopy. Cells (10^5^) were incubated with 10 µg SEVs labelled with PKH67 (green) for 24 h. The supematant of free SEVs-labelled was used as a negative control (NC). Scare bars = 20 μm. CH, Chenghua; LW, Large White; HE, hematoxylin-eosin. The data was calculated using student’s t test. Data are expressed as means ± SEM

Histomorphological analysis revealed that the skin of CH pigs was thicker than that of LW pigs (8.5 mm vs. 3.0 mm) and that the collagen fibres were more densely packed in CH pigs than in LW pigs (Fig. 1A and Additional file 1: Fig. S1A). To examine the effects of SEVs from CHDFs and LWDFs on DFs and skin tissue, we isolated SEVs from the conditioned medium of CHDFs and LWDFs by differential ultracentrifugation and then characterized their morphological characteristics, size distribution, and surface marker expression. Transmission electron microscope (TEM) showed that the isolated SEVs exhibited the typical cup-shaped microscopy (Fig. 1B); in addition, nanoparticle tracking analysis (NTA) revealed that the diameters ranged from 40 to 130 nm, of which the majority were approximately 76 nm (Fig. 1C). Western blot (WB) results showed that surface markers such as CD9, CD63 and CD81 were abundant in these particles (Fig. 1D and Additional file 2: Table S1). Flow cytometric analysis further confirmed the presence of surface markers including CD9 and CD63 (Fig. 1E). The characteristics of these isolated SEVs were consistent with previous studies [25]. Moreover, coincubation of fibroblasts (CHDFs, LWDFs, and HDFs) with CH-SEVs labelled with PKH67 (green) revealed the uptake of these nanoparticles into these cells (Fig. 1F and Additional file 1: Fig. S1B).

### Effects of different SEVs on DFs in vitro

**Fig. 2:**
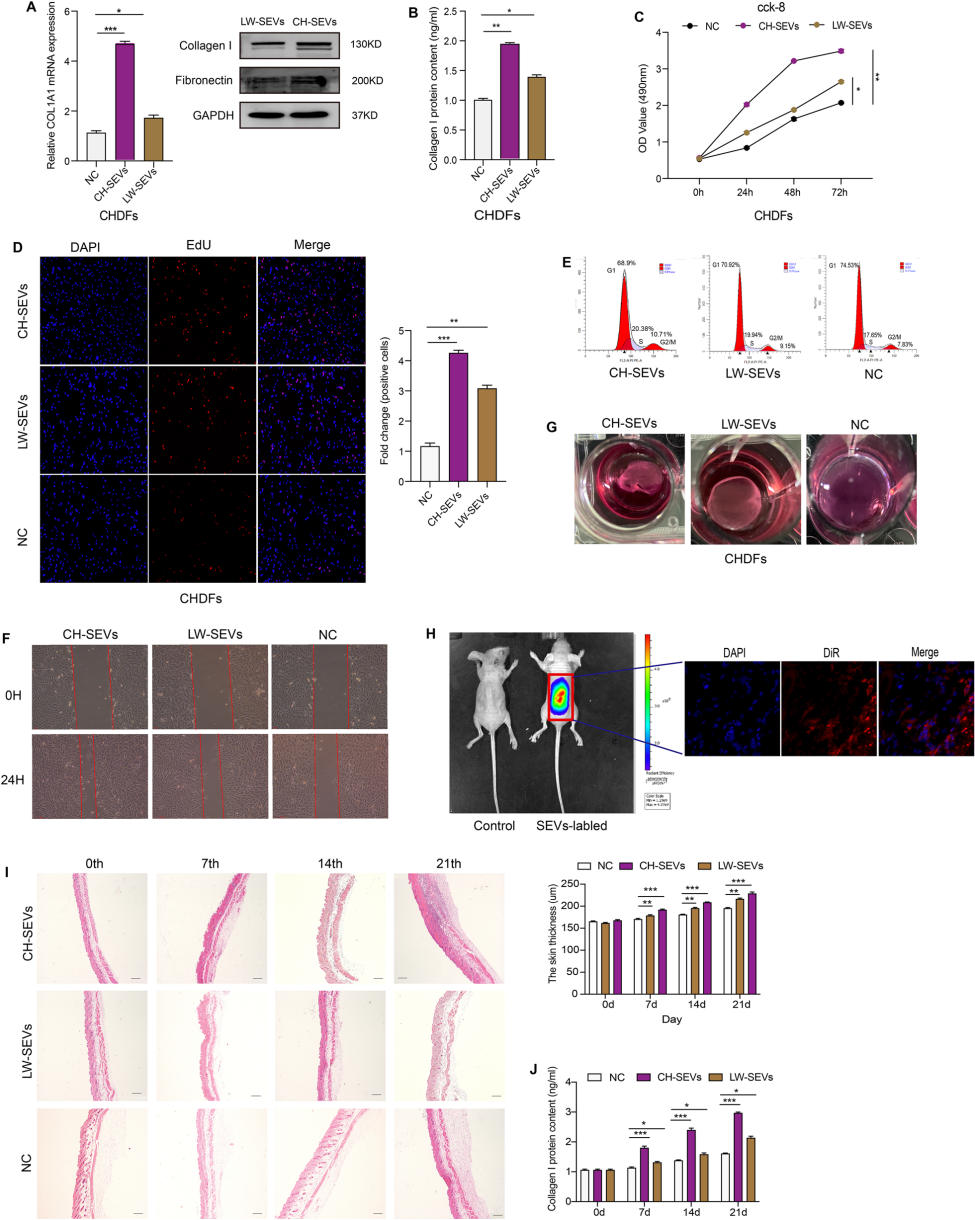
Comparison effect of SEVs from CHDFs and LWDFs in vitro and in vivo. **A** mRNA levels of COL1A1 and WB images of collagen I and fibronectin in CHDFs treated with CH/LW-SEVs. n = 3. **B** Collagen I content of CHDFs treated with CH/LW-SEVs. n = 3. **C** Proliferation of CHDFs treated with CH/LW-SEVs by CCK-8. n = 3. **D** EdU analysis of CHDFs treated with CH/LW-SEVs. n = 3. Scare bars = 100 μm. **E** Cell cycle of CHDFs treated with CH-SEVs and LW-SEVs. Data are the percentage of G1, G2/M, and S. n = 3. **F** Wound recovery area of CHDFs treated with CH-SEVs and LW-SEVs. Objective area is between the two red line. n = 3. **G** Images of collagen gel contraction in CHDFs treated with CH/LW-SEVs. n = 3. **H** Location of CH-SEVs in mouse skin by IVIS imaging system (on the left) and frozen section (on the right). The 100 µL of SEVs labelled with DiR dye were injected by subcutaneous in the buttocks of nude mice. The supematant of free SEVs-labelled was used as a negative control (NC). n = 3. **I** Skin thickness of mice treated with CH/LW-SEVs. n = 3. **J** Collagen I content of mouse skin treated with CH/LW-SEVs. n = 3. Data was calculated using student’s t test and two-way ANOVA followed by Bonferroni’s multiple comparisons test. Data are expressed as means ± SEM; with P* < 0.05, P** < 0.005, P*** < 0.001

To comparatively investigate the effects of SEVs from two different types of pig dermalfibroblasts on the activity of DFs in vitro, three types of DFs (CHDFs, LWDFs, and HDFs) were incubated with CH-SEVs and LW-SEVs, respectively. As collagen type I predominates in the dermis and is responsible for the tensile strength of skin tissue [1], the assessment of its effect on the synthesis of collagen I and fibronectin in DFs was performed by qRT–PCR, WB, and ELISA. After coincubation for 24 h, in all three types of DFs, compared to the negative control (NC) group (PBS), both treatment groups showed a significantly higher expression level of COL1A1 mRNA; moreover the CH-SEV group had a relatively higher expression level than the LW-SEV group. In addition, the WB images for collagen I and fibronectin showed more visually increased bands in the CH-SEV group versus the LW-SEV group (Fig. 2A, Additional file 1: Fig. S2A and Additional file 2: Table S2). The ELISA further revealed that, compared to the NC group, the expression levels of collagen I were upregulated approximately 2-fold for the CH-SEV group and 1.5-fold for the LW-SEV group in all three types of DFs, respectively (Fig. 2B and Additional file 1: Fig. S2B).

DF proliferation was investigated with CCK-8, EdU, and flow cytometry assays. The results of CCK-8 and EdU assays showed that, compared to the NC group, CH-SEV group exhibited a striking ability of cell proliferation, while LW-SEV group showed a slight ability of cell proliferation in all three types of DFs, respectively (Fig. 2C, D, and Additional file 1: Fig. S2C, D). Moreover, the flow cytometry assay further revealed that, compared to the NC group, both treatment groups exhibited a significantly faster cell division progression, while the CH-SEV group had a relatively faster cell division progression than the LW-SEV group, with 9.9–15.6% of the cell in G2/M phase in the CH-SEV group and only 8.4–12.3% of the cell in the G2/M phase in the LW-SEV group in all three types of DFs, respectively (Fig. 2E and Additional file 1: Fig. S2E). In addition, the migration of DFs was investigated with a wound healing assay. After treatment for 24 h, in all three types of DFs, compared to the NC group, both treatment groups showed a significantly faster rate of wound recovery, moreover CH-SEV group showed a relatively faster recovery than LW-SEV group (Fig. 2F, Additional file 1: Fig. S2F, and Additional file 2: Table S3).

In addition, to confirm the contraction ability of SEV-treated fibroblasts, a collagen gel contraction assay was conducted in three types of DFs treated with CH-SEVs and LW-SEVs, respectively. The results showed that, in all three types of DFs, treatment with CH-SEVs and LW-SEVs stimulated collagen gel contraction compared with the NC group, while the CH-SEV group showed a more visibly accelerated contraction than the LW-SEV group (Fig. 2G, Additional file 1: Fig. S2G, and Additional file 2: Table S4). These results indicated that the SEVs originating from CHDFs and LWDFs could stimulate DF activity in vitro, while CH-SEVs were more advantageous in promoting DF proliferation, migration ability, collagen secretion, and collagen gel contraction.

### Effects of injection with different SEVs on skin tissue in mice

To effectively regulate skin tissue, SEVs must penetrate through the epidermis to reach the dermis. Therefore, to realize the biodistribution of the SEVs in mouse skin tissue, we injected approximately 100 µg of purified CH-SEVs labelled with DiR dyes into the dorsum of nude mice. After one day, the fluorescence activity was monitored by the IVIS imaging system; in addition, the image of the frozen section of the mouse skin biopsies showed that the labelled SEVs reached the dermis (Fig. 2H).

Then, to evaluate the efficacy of different SEV treatments on the development of mouse skin, 6-week-old C57 mice received CH-SEVs and LW-SEVs (10^10^ particles/mL) by subcutaneous injection every day for 21 days, respectively. On the 0th, 7th, 14th, and 21st days after treatment, we found that, compared to the NC group (PBS), the thickness of mouse skin was significantly increased in both treatment groups by HE staining, with the CH-SEV group showing a relatively thicker skin than the LW-SEV group (Fig. 2I). Similarly, the ELISA results showed that the collagen I content of mouse skin tissue was significantly upregulated in both treatment groups compared to the NC group, while the CH-SEV group had a relatively higher collagen I content than the LW-SEV group (Fig. 2J). All these data suggested that SEVs derived from both CHDFs and LWDFs could promote skin development, while CH-SEVs were more effective in regulating skin development.

### Identification of enriched microRNAs in CH-SEVs by small RNA sequencing

**Fig. 3:**
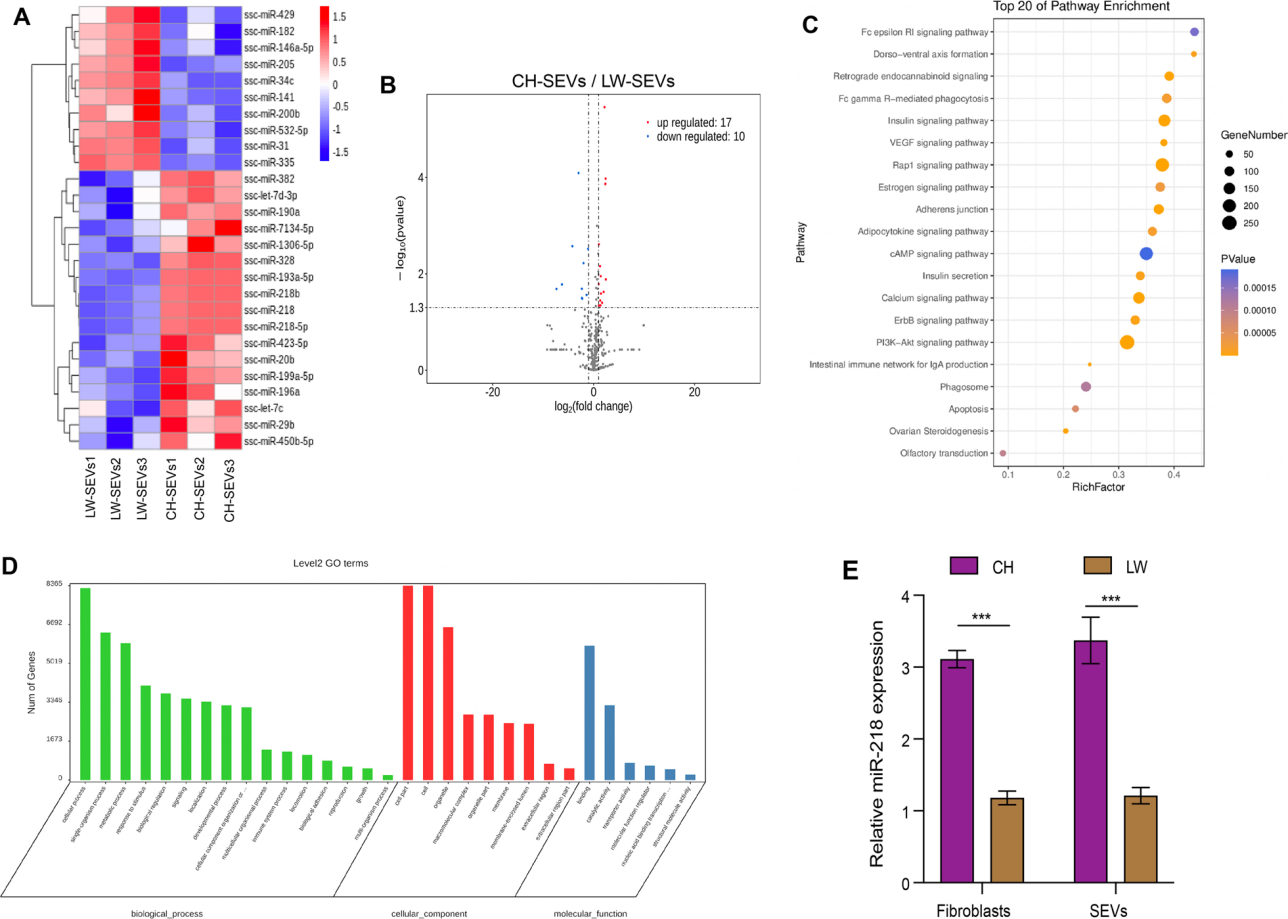
Bioinformatics analysis for differentially expressed miRNAs between CH-SEVs and LW-SEVs. **A** Heatmap of differential miRNAs between CH-SEVs and LW-SEVs. n = 3. Pseudo-colors show expression levels from red (high) to blue (low). **B** Volcano plots of differential miRNAs between CH-SEVs and LW-SEVs. Red and blue plots represent up and down-regulated miRNAs, respectively. n = 3. **C** KEGG for target genes from differential miRNAs. **D** GO for these target genes from differential miRNAs. **E** Expression levels of miR-218 in CH/LW-SEVs, CHDFs, and LWDFs. n = 3. The data was calculated using student’s t test and two-way ANOVA followed by Bonferroni’s multiple comparisons test. Data are expressed as means ± SEM; with P* < 0.05, P** < 0.005, P*** < 0.001

SEVs can mediate cell-to-cell communication and regulate the properties of target cells through some factors, such as proteins, nucleic acids, carbohydrates, and lipids [12], and the effectively specific roles of CH-SEVs on DFs and skin tissue prompted us to further explore the molecular mechanisms of the enriched microRNAs in the SEVs. Therefore, we performed small RNA sequencing of CH-SEVs and LW-SEVs. As a result, we screened a total of 327 miRNAs in the two types of SEVs (Additional file 3: Dataset S1). Among these data, 27 differentially expressed miRNAs were obtained between the CH-SEVs and LW-SEVs (Additional file 3: Dataset S2) and are shown on the heatmap (Fig. 3A) and volcano plots (Fig. 3B). The KEGG and GO analyses showed that these targeted genes for the differentially expressed miRNAs were mainly enriched in some regulatory pathways, such as the Rap1 signalling pathway and PI3K-AKT signalling pathway (Fig. 3C), which regulate cell adhesion, proliferation, and migration [26, 27] and are involved in numerous cellular functions, such as biological regulation and extracellular region (Fig. 3D).

To further explore the potential roles of these differentially expressed miRNAs, we measured their expression levels in two types of SEVs by qRT–PCR (Additional file 2: Table S5). The results showed that miR-218 displayed a relatively high expression level with a greater fold-increase in both CH-SEVs (3.9-fold) and CHDFs (3.2-fold) compared with the LW-SEVs and LWDFs, respectively (Fig. 3E). Coincidentally, a previous study reported that miRNA-218 regulated the ability of TGFβ to induce myofibroblast differentiation in fibroblasts [28]. The results indicated that miRNA-218 might be a CH pig specific regulatory element and play an important role in the development of skin tissue.

### miR-218 targets TGIF2 and activates the TGFβ1-SMAD2/3 pathway to stimulate fibroblast activity and skin development

**Fig. 4:**
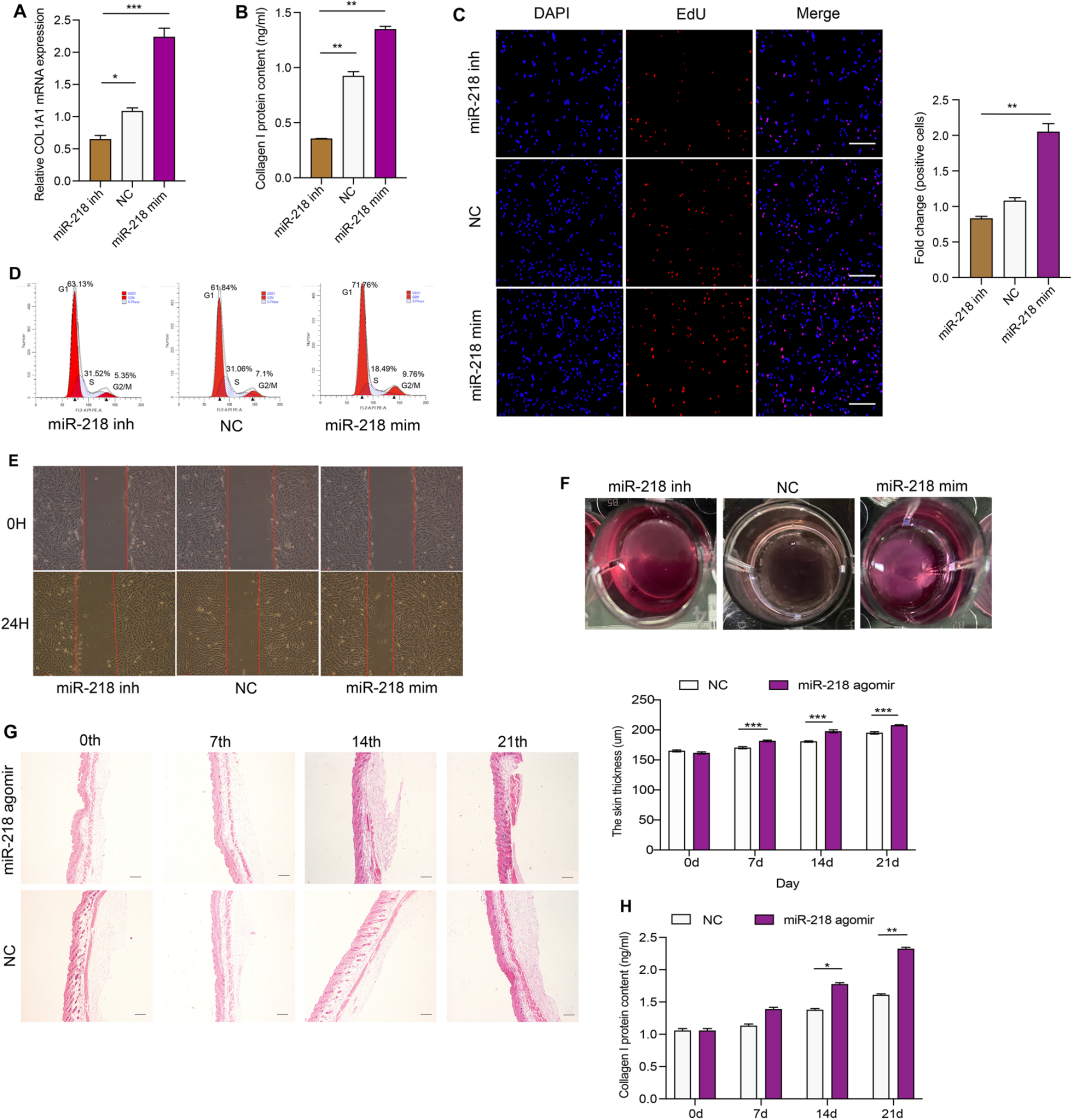
Effect of miR-218 on fibroblast and skin tissue. Expression levels of COL1A1 mRNA (**A**) and collagen I content (**B**) in CHDFs transfected with miR-218. n = 3. **C** Proliferation of CHDFs transfected with miR-218. n = 3. Scare bar = 100 μm. **D** Cell cycle of CHDFs transfected with miR-218. Data are the percentage of G1, G2/M and S. n = 3. **E** Wound recovery area of CHDFs treated with miR-218. Objective area is between the two red lines. n = 3. **F** Images of collagen gel contraction in CHDFs treated with miR-218. n = 3. Skin thickness (**G**) and collagen I content (**H**) of mice injected miR-218 agomir. n = 3. The data was calculated using student’s t test and two-way ANOVA followed by Bonferroni’s multiple comparisons test. Data are expressed as means ± SEM; with P* < 0.05, P** < 0.005, P*** < 0.001

To further identify the potential role of miR-218 in vitro, CHDFs were transfected with miR-218 mimics or inhibitor, and the expected transfection efficiencies were obtained after 24 h (Additional file 1: Fig. S3A). The qRT–PCR and ELISA results showed that, compared to the NC group, the expression levels of collagen I were notably increased after overexpression of miR-218, but the effect was reversed after transfection with the miR-218 inhibitor (Fig. 4A and B). EdU analysis showed that overexpression of miR-218 increased DF proliferation, whereas the opposite results were found after transfection with the miR-218 inhibitor (Fig. 4C). Flow cytometry analysis showed that 9.7% of DFs were at the stage of division after overexpression of miR-218, but only 5.3% of DFs at the stage of division in the inhibitor group (Fig. 4D). Moreover, wound healing analysis showed that DF migration was largely accelerated after treatment with miR-218 mimics, while treatment with miR-218 inhibitor inhibited DF movement (Fig. 4E and Additional file 2: Table S3). Collagen gel contraction assays showed that miR-218 mimics stimulated collagen contraction while the miR-218 inhibitor had an inhibitory effect (Fig. 4F and Additional file 2: Table S4).

To further verify the function of miR-218 mimics in vivo, we injected miR-218 agomir into 6-week-old C57 mice every day for 21 days. On the 0th, 7th, 14th and 21st days after treatment, we found that the thickness of the mouse skin was gradually increased in the miR-218 agomir group compared with the NC group (Fig. 4G); in addition, the ELISA results showed that the skin of mice injected with the miR-218 agomir had a higher collagen I protein content than the NC group (Fig. 4H).

**Fig. 5:**
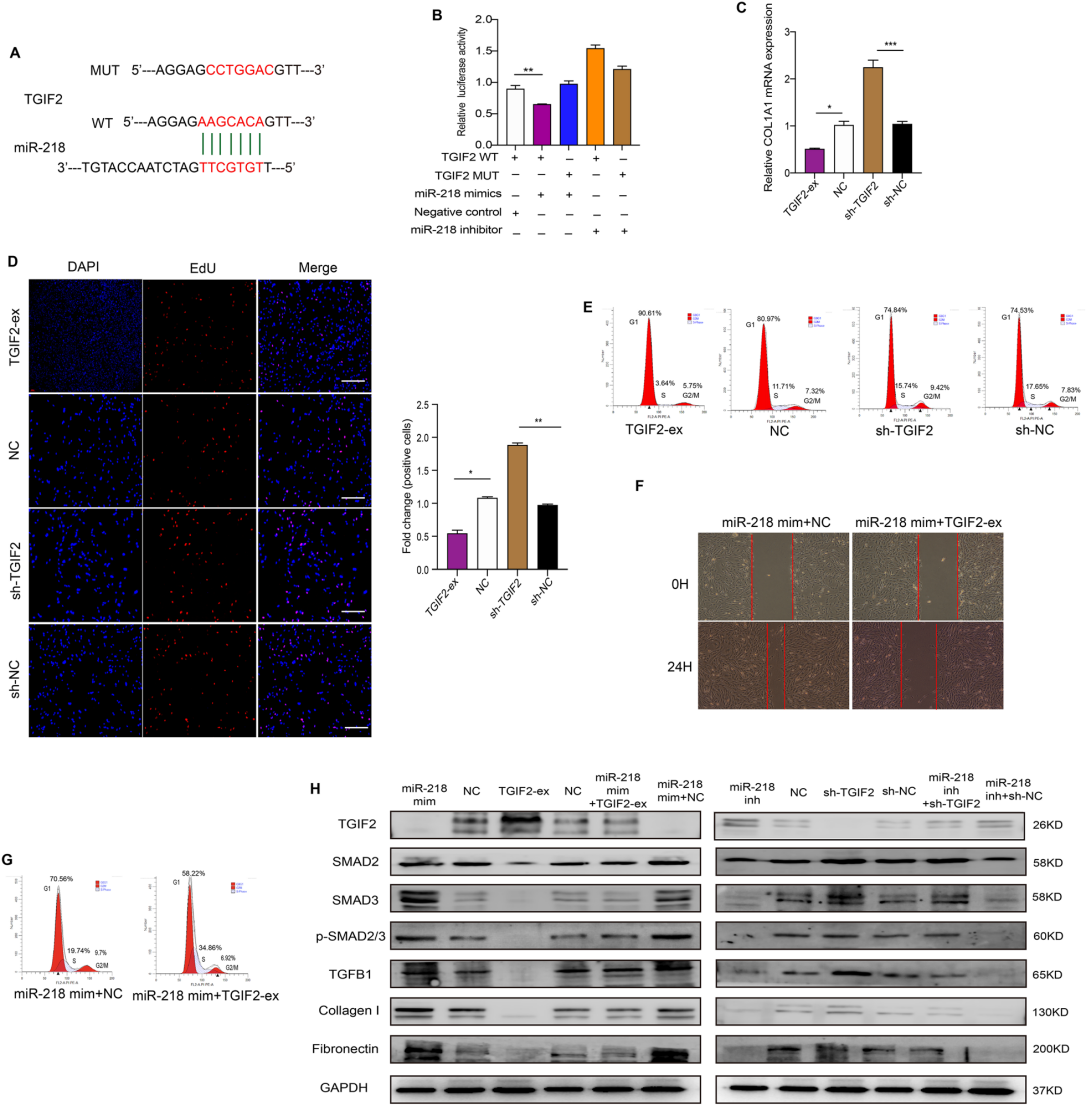
miR-218 targeted TGIF2 and TGIF2 repressed fibroblast activity via TGFβ1-SMAD2/3 pathway. **A** Sequence alignment between TGIF2 and miR-218. **B** Relative luciferase activity of between TGIF2 and miR-218. n = 3. **C** Expression levels of COL1A1 mRNA in CHDFs transfected with TGIF2. n = 3. **D** Proliferation of CHDFs transfected with TGIF2. n = 3. Scare bar = 100 μm. **E** Cell cycle of CHDFs transfected with TGIF2. Data are the percentage of G1, G2/M and S. n = 3. **F** Wound recovery area of CHDFs treated with miR-218 and TGIF2. Objective area is between the two red lines. n = 3. **G** Cell cycle of CHDFs co-treated with miR-218 and TGIF2. Data are the percentage of G1, G2/M and S. n = 3. **H** Protein levels of the related genes in CHDFs. n = 3. The data was calculated using student’s t test and two-way ANOVA followed by Bonferroni’s multiple comparisons test. Data are expressed as means ± SEM; with P* < 0.05, P** < 0.005, P*** < 0.001

TGIF2, the target gene of miR-218, is regarded as a transcription factor inhibitor of the TGFβ pathway [29], which is related to the ability of DFs to synthesize and secrete collagen I [30]. Sequence analysis showed that there is a complementary sequence between the 3′UTRs of the TGIF2 gene and the seed sequence of miR-218 (Fig. 5A). A luciferase reporter assay showed that the luciferase activity was significantly decreased after cotransfection of miR-218 mimics and TGIF2 wild type vectors compared with cotransfection of miR-218 mimics and TGIF2 mutant-type vectors (Fig. 5B). Overexpression of miR-218 significantly downregulated the mRNA level of TGIF2, whereas inhibition of miR-218 significantly upregulated the mRNA level of TGIF2 (Additional file 1: Fig. S3B).

Next, we transfected TGIF2 into CHDFs. After overexpression of TGIF2, the mRNA level of COL1A1 was significantly reduced, as shown by qRT–PCR and DFs proliferation was also significantly suppressed, as shown by EdU and cell cycle assays, but the opposite results were found after knockdown of TGIF2 (Fig. 5C–E). Furthermore, after cotreatment with miR-218 mimics and TGIF2 overexpression vectors, we found that the proliferation and migration of DFs were suppressed by wound healing and cell cycle assays (Fig. 5F, G, and Additional file 2: Table S3).

To evaluate the effect of miR-218 and TGIF2 on the downstream TGFβ1-SMAD2/3 signalling pathway, which is related to the ability of DFs to synthesize and secrete collagen I [30], we transfected miR-218 and TGIF2 into CHDFs and then assessed the protein levels of related genes by WB (Fig. 5H and Additional file 2: Table S6, S7). After transfection of miR-218 mimics, the protein levels of SMAD2, SMAD3, p-SMAD2/3, TGFβ1, collagen I, and fibronectin were significantly upregulated compared to the negative control; similar results were also found after knockdown of TGIF2. However, the opposite results were found after transfection of miR-218 inhibitor or TGIF2 overexpression vectors, respectively. After cotransfection with miR-218 mimics and TGIF2 overexpression vectors, the protein expression levels of these genes were suppressed. The above results indicated that miR-218 might stimulate fibroblast activity and promote skin development, as well as activate the TGFβ1-SMAD2/3 signalling pathway by targeting TGIF2.

### Identification of enriched proteins in CH-SEVs by LC–MS/MS analysis

**Fig. 6:**
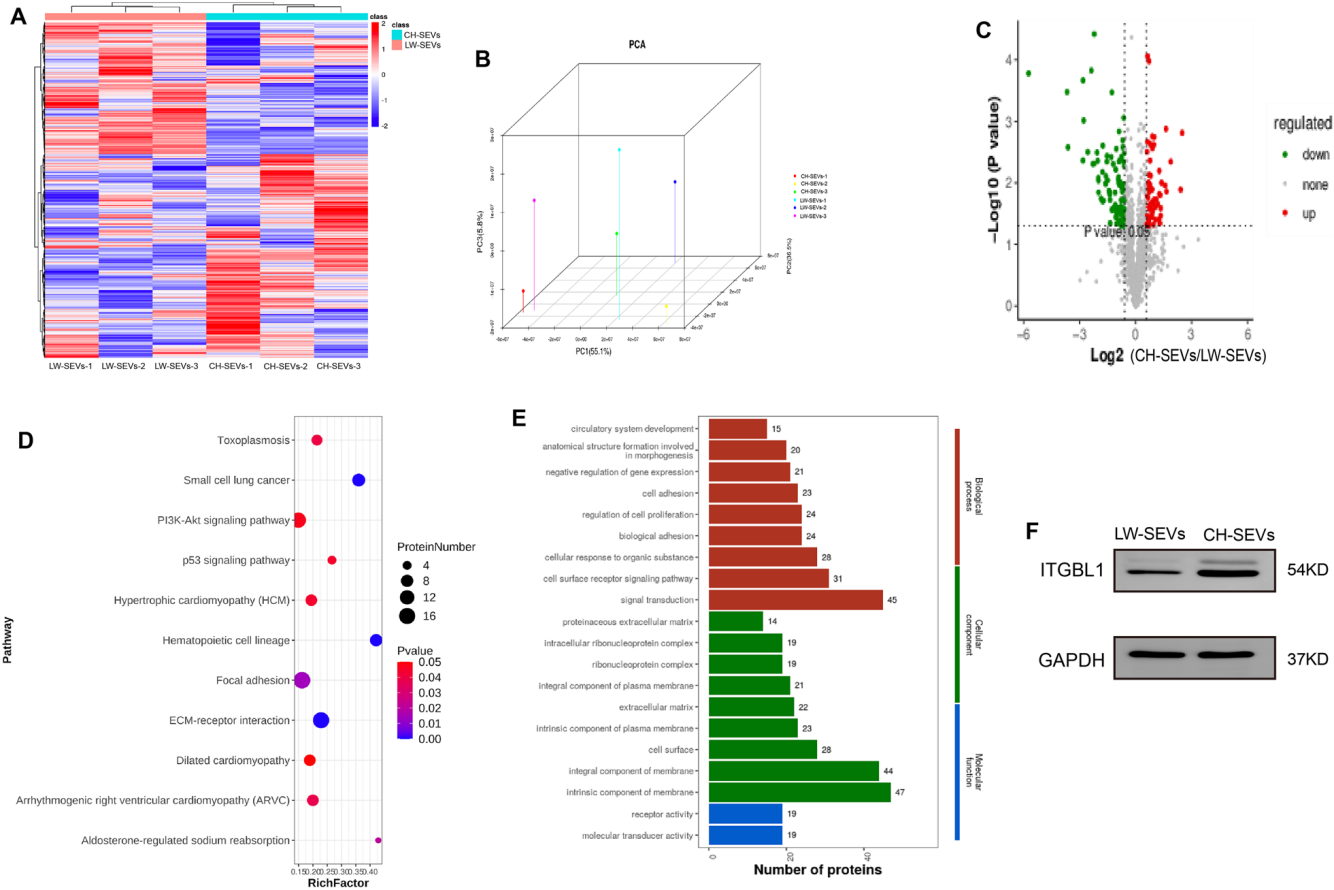
Bioinformatics analysis for differentially expressed proteins between CH-SEVs/LW-SEVs. **A** Heatmap of total proteins between CH-SEVs and LW-SEVs. Pseudo-colors show expression levels from red (high) to blue (low). n = 3. **B** 3D-principal component analysis plot based on expression level of total proteins between CH-SEVs and LW-SEVs. n = 3. **C** Volcano plots of differential proteins between CH-SEVs and LW-SEVs. Red and green plots represent up and down-regulated proteins. n = 3. **D** KEGG for these differential proteins. **E** GO for these differential proteins. **F** Protein levels of ITGBL1 in - CH/LW-SEVs. n = 3. The data was calculated using student’s t test. Data are expressed as means ± SEM

To determine the enriched proteins in CH-SEVs, LC–MS/MS analysis was performed on both CH-SEVs and LW-SEVs. As a result, we screened a total of 1613 proteins (Additional file 3: Dataset S3), and their expression pattern is shown in Fig. 6A and B, which proved the discrepancy between CH-SEVs and LW-SEVs. Among these data, 151 differentially expressed proteins were obtained between CH-SEVs and LW-SEVs (Fig. 6C and Additional file 3: Dataset S4). The KEGG and GO analyses showed that these differentially expressed proteins were mainly enriched in some regulatory pathways, such as the focal adhesion and ECM-receptor interaction pathways (Fig. 6D) and were involved in numerous cellular functions, such as signal transduction, integral component of membrane, and intrinsic component of membrane (Fig. 6E). Interestingly, considering the top 15 upregulated proteins with a fold change and the significant pathway of focal adhesion and ECM-receptor interaction, we found that the ITGBL1 protein could play an important role in fibroblasts and skin tissue. Coincidentally, a previous study reported that ITGBL1 was a key upstream regulator of liver fibrosis via interactions with TGFβ [31]. In addition, WB assays showed that the ITGBL1 protein was remarkably enhanced in CH-SEVs compared with LW-SEVs (Fig. 6F and Additional file 2: Table S2).

### ITGBL1 promotes fibroblast activity and skin development by regulating the TGFβ1-SMAD2/3 pathway

**Fig. 7:**
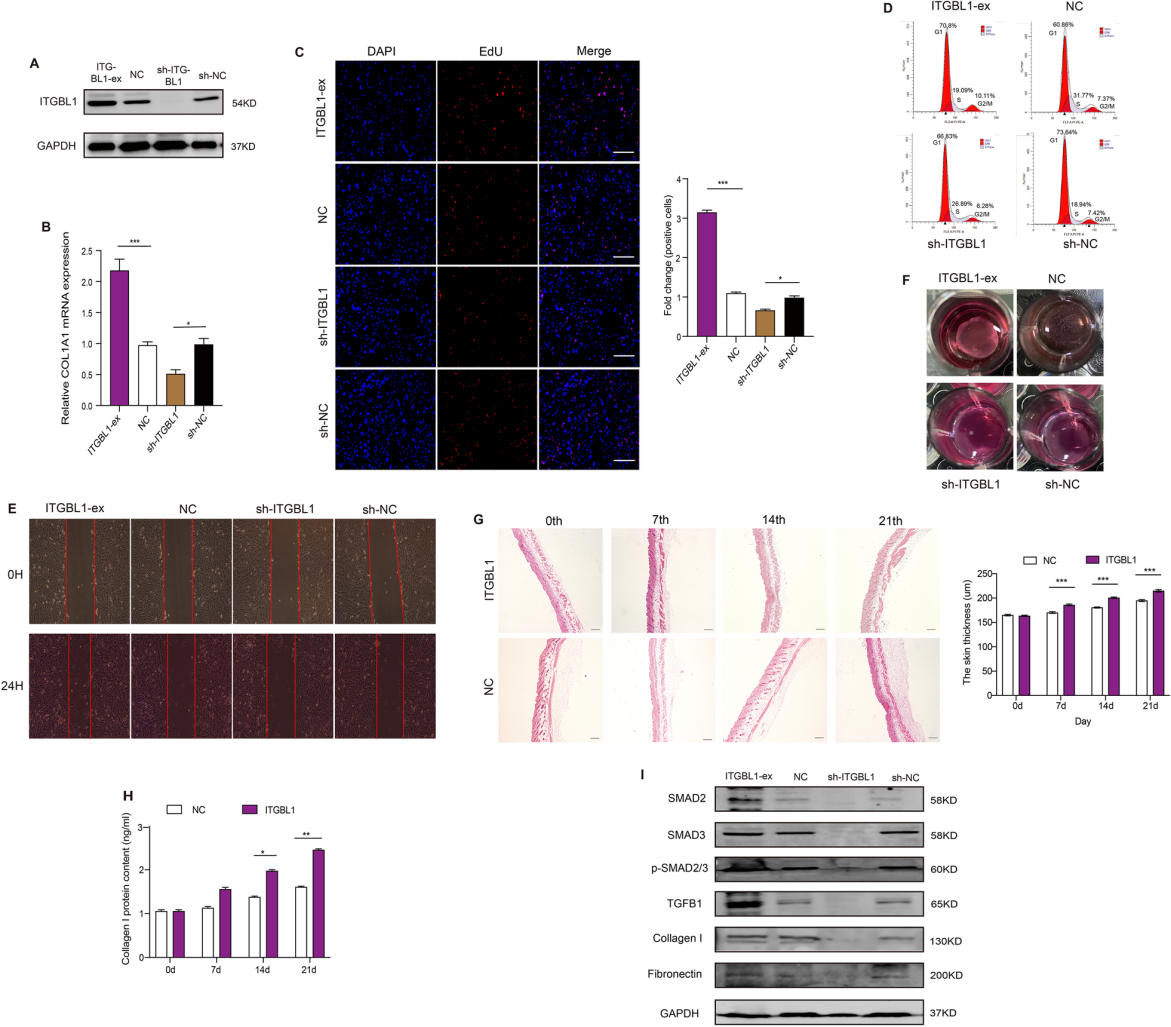
Effect of ITGBL1 protein on fibroblast and skin tissue. **A** Protein levels of ITGBL1 in CHDFs transfected with ITGBL1. n = 3. **B** mRNA levels of COL1A1 in CHDFs transfected with ITGBL1. n = 3. **C** Proliferation of CHDFs transfected with ITGBL1. n = 3. Scare bar = 100 μm. **D** Cell cycle of CHDFs transfected with ITGBL1. Data are the percentage of G1, G2/M and S. n = 3. **E** Wound recovery area of CHDFs treated with ITGBL1. Objective area is between the two red lines. n = 3. **F** Images of collagen gel contraction in CHDFs treated with ITGBL1. n = 3. Skin thickness (**G**) and collagen I content (**H**) of mice injected ITGBL1 vectors. n = 3. **I** Protein levels of the related genes in CHDFs treated with ITGBL1. n = 3. Data was calculated using student’s t test and two-way ANOVA followed by Bonferroni’s multiple comparisons test. Data are expressed as means ± SEM; with P* < 0.05, P** < 0.005, P*** < 0.001

To verify the function of ITGBL1 in vivo, we carried out a transfection experiment for ITGBL1 in CHDFs and then obtained the expected transfection efficiencies by WB (Fig. 7A and Additional file 2: Table S8). The qRT–PCR results showed that the mRNA level of COL1A1 was notably increased after overexpression of ITGBL1, but the effect was reversed after transfection with sh-ITGBL1 (Fig. 7B). Using EdU and cell cycle assays, we found that overexpression of ITGBL1 significantly promoted DF proliferation, whereas knockdown of ITGBL1 had a contradictory effect (Fig. 7C and D). Wound healing analysis showed that DF migration was largely accelerated after treatment with ITGBL1 overexpression vectors, while treatment with sh-ITGBL1 inhibited DF movement (Fig. 7E and Additional file 2: Table S3). Collagen gel contraction assays showed that overexpression of ITGBL stimulated protein contraction but knockdown of ITGBL1 had the opposite effect (Fig. 7F and Additional file 2: Table S4).

To further confirm the function of ITGBL1 in vivo, we constructed an ITGBL1 vector with pcDNA3.1 and injected it into 6-week-old C57 mouse skin tissue every day for 21 days. On the 0th, 7th, 14th and 21st days after treatment, we found that the skin thickness of mice treated with ITGBL1 vector was gradually increased compared with that of negative control mice (PBS) through HE staining (Fig. 7G); in addition, the ELISA results showed that the skin of mice injected with the ITGBL1 vector had a higher collagen I protein content than the negative control group (Fig. 7H).

Previous studies showed that ITGIBL1 protein could promote migration and invasion in hepatocellular carcinoma cells by stimulating the TGFβ/SMAD signalling pathway through interaction with TGFβ1 [32, 33]. To determine whether ITGBL1 can act as an activator downstream of the TGFβ1-SMAD2/3 signalling pathway, we transfected ITGBL1 into CHDFs and then assessed the protein expression levels of related genes by WB (Fig. 7I and Additional file 2: Table S8). After transfection with the ITGBL1 plasmid, the protein levels of SMAD2, SMAD3, p-SMAD2/3, TGFβ1, collagen I, and fibronectin were significantly upregulated compared to those in the negative control (pcDNA3.1), whereas the opposite results were also found after treatment with sh-ITGBL1. The above results suggested that ITGBL1 might stimulate fibroblast activity by interacting with the TGFβ1-SMAD2/3 signalling pathway and promote skin development.

## Discussion

Dermal fibroblasts are essential for skin regeneration and damage repair because they can produce abundant collagens and cytokines that are primary constituents of the dermal extracellular matrix [31]. Although autologous HDF injections are capable of improving facial contour defects and creating a continuous protein repair system to reduce wrinkle formation, DFs gradually lose their capacity to proliferate and synthesize collagen with ageing. Recently, SEVs derived from HDFs have been reported to be effective materials to protect and repair skin damage, because they can ameliorate skin-ageing [17, 18] and promote cutaneous wound healing [19, 20], which indicates that SEVs from HDFs may be potential materials to protect and repair skin damage. In this research, using pigs as a model of humans, we isolated SEVs from pig DFs and demonstrated that treatment with SEVs derived from pig DFs can promote fibroblast proliferation, migration, collagen contraction, and synthesis of collagen I and fibronectin; similarly, injections of SEVs derived from pig DFs were also observed increase the thickness and collagen I content of mouse skin in vivo. Moreover, the effect of SEVs derived from pigs was more advantageous for cell activity in HDFs. Thus far, our results indicated that SEVs derived from pig DFs and HDFs play a vital role in accelerating fibroblast activity and skin development.

Notably, the cell state of DFs can affect their biological functions. For example, previous research found that old HDFs, which lose the fibroblast states present in young skin, were no longer clearly demarcated and showed not only reduced expression of genes involved in the formation of the extracellular matrix but also the appearance of adipogenic traits [34]. Similarly, SEVs derived from primary fibroblasts of young humans can ameliorate certain biomarkers of senescence in old fibroblasts and in a variety of tissues in old mice [18]. Moreover, compared to the SEVs derived from the monolayer culture of HDFs (2D HDF-SEVs), the SEVs-derived from three-dimensional spheroids (3D HDF-SEVs) were more effective at regulating dermal fibroblast proliferation, migration, and protein expression, thus reducing skin ageing [17]. Here, we found that SEVs derived from DFs of CH pigs with superior skin thickness were more effective than SEVs derived from LWDFs at stimulating fibroblast activity in vitro and promoting mouse skin development in vivo, which may partially account for the development of superior skin thickness in CH pigs.

The ability of DFs to synthesize and secrete collagen I is primarily regulated by the TGFβ pathway [1, 35]. DFs can produce many cytokines and growth factors for skin cells, and these bioactive molecules may be packaged by SEVs and delivered to target cells to prompt the activities of recipient cells [12, 36, 37]. Here, we identified an enriched miRNA in CH-SEVs, miRNA-218, which promoted fibroblast activity and skin development and revealed that the activation of the miR-218-TGIF2-TGFβ-SMAD2/3 pathway was involved in the positive effects of the DF-SEVs. Therefore, the increased miR-218 in CH-SEVs could prompt the development of superior skin thickness in CH pigs via the TGFβ-SMAD2/3 pathway. Similarly, a previous study revealed that miRNA-218 can regulate the ability of TGFβ to induce myofibroblast differentiation in fibroblasts via cezanne/FAK; compared with gingival fibroblasts, the dermal fibroblasts showed increased expression levels of miR-218 and resulted in the ability of TGFβ to induce α-SMA in fibroblasts [28]. In addition, several studies reported that TGIF2, the target gene for miR-218, is a SMAD transcriptional corepressor that negatively regulates TGFβ-activated gene expression in vertebrates [38, 39] and plays a role in spermiogenesis and folliculogenesis [29].

Moreover, we identified an enriched active protein known as ITGBL1 in CH-SEVs, which promoted fibroblast activity and skin development by stimulating the TGFβ/SMAD2/3 signalling pathway. Similarly, in liver cells, a previous study revealed that ITGBL1 promotes migration and invasion in hepatocellular carcinoma cells by stimulating the TGFβ/SMADs signalling pathway through interaction with TGFβ1 [32]; moreover, ITGBL1 was demonstrated to be as a key upstream regulator of liver fibrosis via interactions with TGFβ [40]. In addition, one study found that in lung cells, ITGBL1 is a target of miR-576-5p and promotes non-small-cell lung cancer (NSCLC) cell migration and invasion through Wnt/PCP signalling [41]; moreover, ITGBL1 plays a role in the pulmonary fibrosis process through the positive feedback of TGFβ1-lncITPF-ITGBL1 [42]. Interestingly, our results indicate that ITGBL1 could be activated by miR-218 via the TGFβ1/SMAD pathway. Here, the increased ITGBL1 in CH-SEVs could prompt the development of superior skin thickness in CH pigs via the TGFβ-SMAD2/3 pathway.

This study revealed that CH-SEVs can effectively stimulate fibroblast activity and promote skin development. Enriched cargos in CH-SEVs, namely miRNA-218 and the ITGBL1 protein, play an important role in stimulating fibroblast activity via activation of the downstream TGFβ1-SMAD2/3 signalling pathway and promoting skin development. These results indicate that SEVs from CH pig dermal fibroblasts can effectively stimulate fibroblast activity and skin development and have the potential to protect and repair skin damage.

## Materials and methods

### Skin phenotype measurements

The total skin thickness from 20 pigs with similar weight at approximately 100 kg per breed was measured with a Vernier calliper, and all pig skin was subjected to Sirius red and haematoxylin-eosin staining; moreover, all experimental mouse skin was also stained with haematoxylin-eosin. All skin dermal thicknesses were measured according to a previously described method [3, 43].

### Cell culture and small extracellular vesicle characterization

The pig primary fibroblasts were separated from the skin tissues of 7-day-old CH and LW pigs. The skin tissues were digested by dispase II and collagenase I. Then, the cells were seeded into T-175 flasks containing DMEM supplemented with 15% foetal bovine serum (FBS) (Gibco, New York, USA). Then, after the cell density reached 70% confluence, we discarded the conditioned medium and used DMEM supplemented with 2% free exosome foetal bovine serum in place of 15% FBS to culture fibroblasts. The fibroblast conditioned medium was collected after 48 h. Then, SEVs were isolated according to previous methods [44], and the supernatant was ultracentrifuged three times at 120,000×*g* for 2 h at 4 °C by TYPE 90 Ti (Beckman Coulter, USA). The pellets were resuspended in phosphate buffer saline (PBS) and stored at − 80 °C until subsequent analysis. Morphological analysis of SEVs was performed by transmission electron microscopy (Hitachi, Japan), and SEVs were concentrated at 10^10^ particles/mL for Nanoparticle Tracking Analysis (Nanosight NS300, Amesbury, UK) using an NTA2.1 Analytical Software. The surface marker proteins CD9/CD63/CD81 were assessed by western blotting. Moreover, SEVs (10^10^ particles/mL) were incubated with magnetic microbeads coated with CD9 or CD63 antibodies, and those antibodies that attached to the SEVs were analysed by flow cytometry. Human dermal fibroblasts (DFs) and 293T cells purchased from the Cell Bank of Chinese Academy of Sciences were cultured with DMEM containing 10% fetal bovine serum in an incubator at 37 °C with 5% CO_2_. Cell lines were tested and did not exist for mycoplasma contamination.

### Labelling of small extracellular vesicles with PKH67/DiR

The SEVs were labelled with PKH67 (green) /DiR (red) fluorescent kits based on the instructions. In briefly, 100 µL of SEVs was added to 5 µL of PKH67/DiR (5 µM) and incubated at 37 °C for 10 min in the dark. Then, the reaction was stopped by adding 1 mL of free exosome bovine serum for 5 min and ultracentrifuged once at 120,000×*g* for 2 h at 4 °C after adding 4 mL of PBS. The labelled SEVs were resuspended in PBS and then were captured using an Olympus IX53 microscope (Olympus; Tokyo, Japan) and stored at 4 °C until further study.

### Isolation of SEV RNA, construction of the library, and RNA sequencing

Total SEV RNA was extracted from the 6 samples (three biological replications per breed SEVs) using RNA extraction reagent according to the manufacturer’s instructions. The purity and concentration of the SEV RNAs were assessed using an Agilent 2100 Bioanalyzer. The small RNA library was constructed by Guangzhou Huayin Health Medical Co., Ltd. Library preparation with fragmentation gel (18–30 nt), ligation of 3′ and 5′ adapters, reverse transcription, PCR amplification and gel recycling were performed. Finally, the libraries were sequenced on an Illumina Nova-seq 6000 following the manufacturer’s recommendations.

### Small RNA sequencing data analysis

High-quality clean reads were obtained by removing the reads, including adapter, polyA and low-quality reads, for subsequent analysis. The known miRNAs were obtained by Bowtie2 (2.2.8) software, which were mapped to ensemble release 99 for each sample. For the small RNA data, the transcripts per million (TPM) were calculated for each sample. The differentially expressed miRNAs were analysed using the edgeR package running in the R programming environment. Differential expression, as determined by a p value < 0.05 and | fold change | > 1, was characterized in two different SEVs. The mRNAs targeted by these miRNAs were functionally analysed by the public GO and KEGG databases.

### Detection of protein by RPLC–MS/MS and data analysis

The SEV protein was digested using a filter-aided sample preparation method [45]. The polypeptide was obtained and analysed by reversed-phase liquid chromatography-tandem mass spectrometry (RPLC–MS/MS). Finally, the mass spectrometry data were obtained using an Orbitrap Fusion Lumos Mass Spectrometer (Thermo Scientific). We used the Uniprot porcine protein sequence database and integrated the DIA mass spectrometry data by Spectronaut Pulsar (Biognosys) software for protein identification and quantitative information. The protein identification mode was DirectDIA with the threshold value of proteins PSM FDR < 0.01, and protein FDR < 0.01. The criteria of fold change > 1.5 and P < 0.05 were used as the screening threshold for differentially expressed proteins. Proteins functional was analysed by the GO and KEGG databases.

### Cell treatment, proliferation, migration and collagen production

When the fibroblast density reached 70–80% confluence, the cell medium was changed to DMEM containing the ITGBL1 and TGIF2 plasmid vectors (200 ng/µL), negative control (pcDNA3.1, 200 ng/µL), sh-ITGBL1 and sh-TGIF2 (500 ng/µL), sh-NC (500 ng/µL), double-stranded miR-218 mimics, or miR-218 inhibitor and negative control (50 µM) for transfection with Lipofectamine 3000 (Thermo Fisher Scientific) for 8 h according to the manufacturer’s instructions. Additionally, the fibroblasts were treated with DMED with CH/LW-SEVs (10^10^ particles/mL) for 24 h as well as a negative control (NC, PBS). Subsequently, proliferation of the cells (10^3^) was assessed at 0 h, 24 h, 48 and 72 h using CCK-8 (Beyotime, Shanghai, China), and EdU (Beyotime, Shanghai, China) staining was performed according to the manufacturer’s protocol. Then, the cell resuspension solution was assessed by flow cytometry (Beckman Coulter, USA) to obtain the ratio data for the different stages of the cell cycle. Moreover, cell migration was assessed by a wound healing assay. In addition, the expression levels of related genes were determined by qRT–PCR and WB. The collagen I content was assessed using ELISA kit (EIAab, Wuhan, China).

### Collagen gel contraction assay

The 3D collagen gel contraction assay was performed with Cell Contraction Assay Kit (Cell Biolabs Inc., CA, USA) according to the manufacturer’s protocol. The cells (10^6^) treated with CH-SEVs, LW-SEVs, miR-218 mimics, negative control or TGIBL1 were mixed with cold Collagen Gel Working Solution. Then, 0.5 mL of a mixture of cells and collagen was transferred into 24 well plates, which were cultured for 1 h at 37 ºC with 5% CO_2_. Then, 0.8 mL of 10% FBS culture medium was added to each well and incubated for 48 h after that. Collagen contraction was initiated by gently releasing the gel from the sides of platelets, after the cells were treated with 10 mM BDM contraction mediators. The collagen contraction gel was obtained, and the areas were measured using ImageJ.

### Luciferase reporter assay

The corresponding sequences of TGIF2-WT/Mut were inserted into psiCHECK-2 vectors (Sangon, Shanghai, China). Approximately 10^4^ 293T cells were cotransfected with TGIF2-WT/Mut and miR-218 mimics/inhibitor using Lipofectamine 3000. The luciferase activity of each group was measured by the GloMax 20/20 Luminescence Detector based on the dual-luciferase reporter assay instructions.

### Animal model

The 6-week-old female C57 mice and 8-week-old nude mice were obtained from Chengdu Dashuo Biotechnology Company. 100 µg/mL of CH-SEVs (n = 3), 100 µg/mL of LW-SEVs (n = 3), 5 μm of miR-218 agomir (n = 3), 20 µg/g of pcDNA3.1-ITGBL1 plasmid vector (Hanbio, Shanghai, China) (n = 3) or 100 µL of PBS as a negative control (NC) groups (n = 3) were stably injected in the buttocks of the mice every day. On the 0th, 7th, 14th, and 21st days, the mice were sacrificed, and skin tissues were obtained at the hip for related analysis.

### Statistical analysis

Statistical testing was conducted with GraphPad Prism. The data are shown as the mean ± SEM for one group. The differences between groups were calculated using Student’s t test and two-way ANOVA followed by Bonferroni’s multiple comparisons test. P < 0.05 was considered to be significant, with *P < 0.05, **P < 0.01, and ***P < 0.001.

Some detailed materials and methods are provided in supplementary materials and methods (Additional file 4).

## Supplementary Information

**Additional file 1**: **Fig S1.** Characterization of skin thickness and SEVs from DFs; **Fig S2.** The effect of SEVs from CHDFs and LWDFs on LWDFs/HDFs; **Fig S3.** The expression levels of miR-218 and TGIF2 in CHDFs.

**Additional file 2**: **Table S1.** The quantitative WB data of SEVs surface marker genes in screening cells and SEVs. **Table S2.** The quantitative WB data in fibroblast treated with CH/LW-SEVs. **Table S3.** The wound healing area in fibroblasts treated with CH/LW-SEVs and related genes. **Table S4.** The collagen gel contraction values in fibroblasts treated with CH/LW-SEVs and related genes. **Table S5.** The fold change of total differential miRNAs by RNA–seq and qRT–PCR. **Table S6/S7.** The quantitative WB data in CHDFs transfected with miR-218 and TGIF2. **Table S8.** The quantitative WB data in CHDFs transfected with ITGBL1.

**Additional file 3**: **Dataset S1.**The total miRNAs in CH-SEVs and LW-SEVs. **Dataset S2.** The differential miRNAs between CH-SEVs and LW-SEVs. **Dataset S3.**The total proteins in CH-SEVs and LW-SEVs. **Dataset S4.**The differential proteins between CH-SEVs and LW-SEVs.

**Additional file 4.** The detailed materials and methods are provided in supplementary materials and methods and include as follows: TEM, NTA, flow cytometry, RPLC-MS/MS, validation of genes by qRT–PCR, SEVs internalization, cell counting kit-8 assay, 5-Ethynyl-2′-deoxyuridine assay, ELISA assay, wound healing assay, cell cycle analysis, western blot analysis, and animal for SEVs labelling.

## Data Availability

All data and materials about this study are included in these additional files.
