# Supplementary material for: Small extracellular vesicles derived from dermal fibroblasts promote fibroblast activity and skin development through carrying miR-218 and ITGBL1
Source: J Nanobiotechnology. 2022 Jun 22;20:296. doi: 10.1186/s12951-022-01499-2 (PMC9215004; doi:10.1186/s12951-022-01499-2)
Supplement: Supplementary file 1 — Additional file 1: Fig S1. Characterization of skin thickness and SEVs from DFs; Fig S2. The effect of SEVs from CHDFs and LWDFs on LWDFs/HDFs; Fig S3. The expression levels of miR-218 and TGIF2 in CHDFs. [file 12951_2022_1499_MOESM1_ESM.docx]

**Additional file 1. Supplementary Figure**


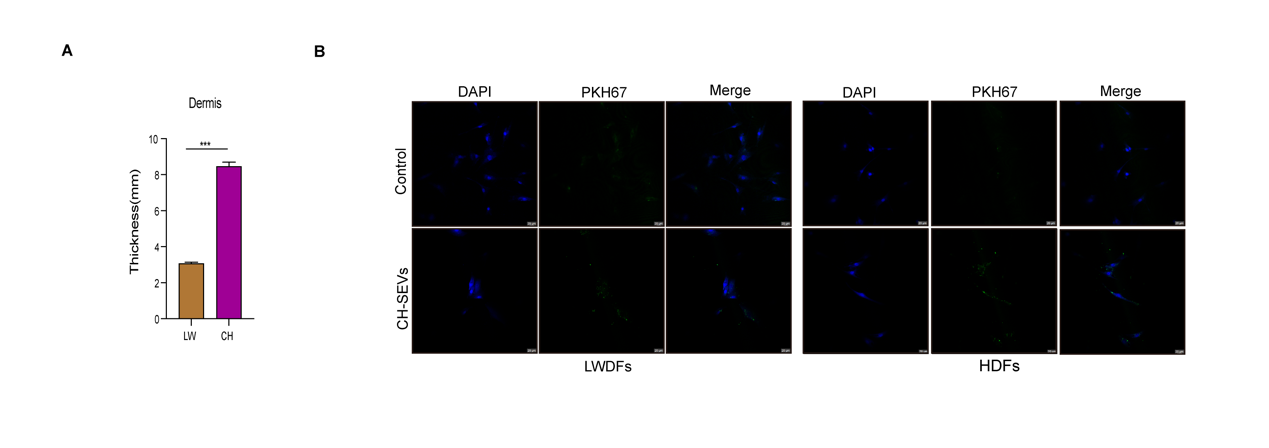


**Figure S1. Characterization of skin thickness and SEVs from DFs.** (A) Dermis thickness of CH and LW pigs. n = 20 per breed. (B) Images of location between CH-SEVs and LWDFs/HDFs by confocal microscopy. Cells (10^5^) were incubated with 10 µg of SEVs labelled with PKH67 (green) for 24 h in confocal dish. The supematant of free SEVs-labelled was used as a control. Scare bars = 20 μm. The data was calculated using student’s t test and two-way ANOVA followed by Bonferroni’s multiple comparisons test. Data are expressed as means ± SEM; with P*** < 0.001.


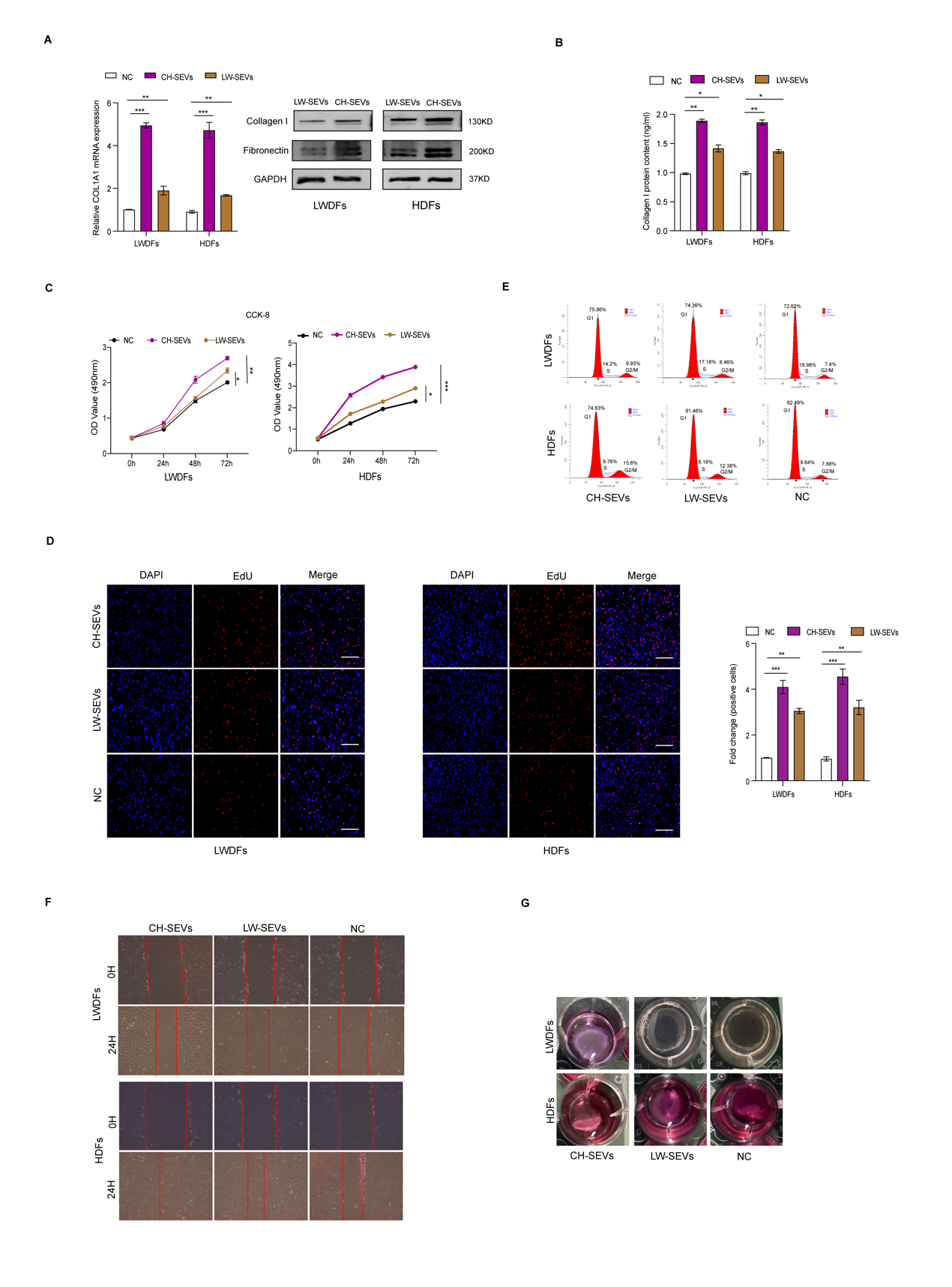


**Figure S2. The effect of SEVs from CHDFs and LWDFs in LWDFs/HDFs.** (A) mRNA levels of COL1A1 and WB images of collagen I and fibronectin in LWDFs/HDFs treated with CH/LW-SEVs. n = 3. (B) Collagen I content of LWDFs/HDFs treated with CH/LW-SEVs. n = 3. (C) Proliferation of LWDFs/HDFs treated with CH/LW-SEVs by CCK-8. n = 3. (D) EdU analysis of LWDFs/HDFs treated with CH/LW-SEVs. n = 3. Scare bars = 100 μm. (E) Cell cycle of LWDFs/HDFs treated with CH/LW-SEVs. Data are the percentage of G1, G2/M, and S. n = 3. (F) Wound recovery area of LWDFs/HDFs treated with CH/LW-SEVs. Objective area is between the two red lines. n = 3. (G) Images of collagen gel contraction in LWDFs/HDFs treated with CH/LW-SEVs. n = 3. The data was calculated using student’s t test and two-way ANOVA followed by Bonferroni’s multiple comparisons test. Data are expressed as means ± SEM; with P* < 0.05, P** < 0.005, P*** < 0.001.


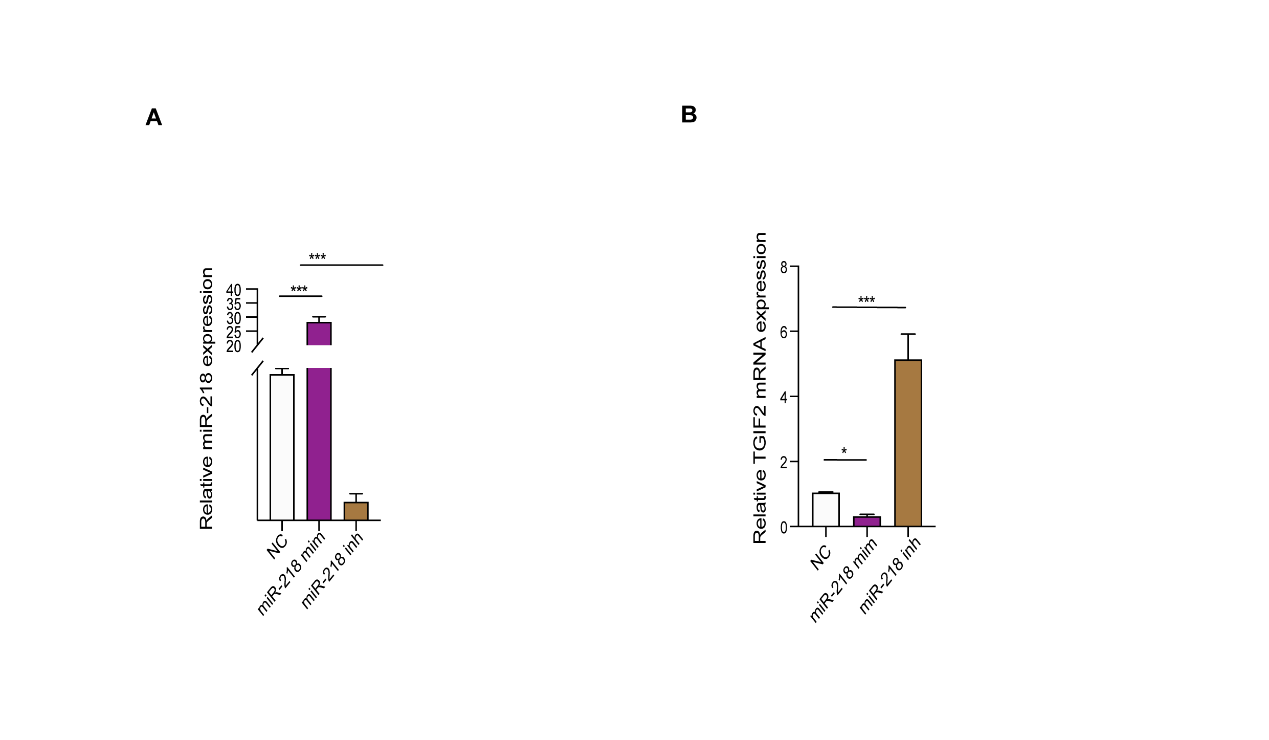


**Figure S3.** **The expression levels of miR-218 and TGIF2 in CHDFs.** (A) Transfection efficiency of miR-218 in CHDFs. n = 3. (B) Expression levels of TGIF2 in CHDFs transfected miR-218. n = 3. The data was calculated using student’s t test and two-way ANOVA followed by Bonferroni’s multiple comparisons test. Data are expressed as means ± SEM; with P* < 0.05, P*** < 0.001.
