# Supplementary material for: Small extracellular vesicles derived from dermal fibroblasts promote fibroblast activity and skin development through carrying miR-218 and ITGBL1
Source: J Nanobiotechnology. 2022 Jun 22;20:296. doi: 10.1186/s12951-022-01499-2 (PMC9215004; doi:10.1186/s12951-022-01499-2)
Supplement: Supplementary file 2 — Additional file 2: Table S1. The quantitative WB data of SEVs surface marker genes in screening cells and SEVs. Table S2. The quantitative WB data in fibroblast treated with CH/LW-SEVs. Table S3. The wound healing area in fibroblasts treated with CH/LW-SEVs and related genes. Table S4. The collagen gel contraction values in fibroblasts treated with CH/LW-SEVs and related genes. Table S5. The fold change of total differential miRNAs by RNA–seq and qRT–PCR. Table S6/S7. The quantitative WB data in CHDFs transfected with miR-218 and TGIF2. Table S8. The quantitative WB data in CHDFs transfected with ITGBL1. [file 12951_2022_1499_MOESM2_ESM.docx]

**Additional file 2. Supplementary Table**

| **Table S1. The quantitative WB data of SEVs surface marker genes**  **in screening cells and SEVs** | | | | |
| --- | --- | --- | --- | --- |
| Genes | CH-cells | CH-SEVs | LW-cells | LW-SEVs |
| CD9 | 0.029 | 0.070 | 0.043 | 0.048 |
| CD63 | 0.039 | 0.070 | 0.059 | 0.090 |
| CD81 | 0.002 | 0.070 | 0.000 | 0.010 |
| The first row is sample name; the first column is gene symbol.  The gray values are calculated basing on the quantization of bands intensities; the relative values are obtained by the ratio that the objective genes gray values divided by GAPDH (as loading control) gene gray values (such as CD9/GAPDH) in the same samples. | | | | |

| **Table S2. The quantitative WB data in fibroblasts treated with CH/LW-SEVs** | | | | | | |
| --- | --- | --- | --- | --- | --- | --- |
| Genes | CHDFs | | LWDFs | | HDFs | |
|  | LW-SEVs | CH-SEVs | LW-SEVs | CH-SEVs | LW-SEVs | CH-SEVs |
| Collagen I | 0.480 | 0.899 | 0.315 | 0.766 | 0.596 | 0.976 |
| Fibronectin | 0.282 | 0.388 | 0.027 | 0.321 | 0.519 | 0.814 |
| ITGBL1 | 0.187 | 0.442 | - | - | - | - |
| The first row is the cell type of fibroblasts; the second row is treatment group; the first column is gene symbol. | | | | | | |

| **Table S3. The wound healing area in fibroblasts treated with**  **CH/LW-SEVs and relative genes** | | | |
| --- | --- | --- | --- |
| Treatment group | CHDFs | LWDFs | HDFs |
| CH-SEVs | 0.027 | 0.035 | 0.012 |
| LW-SEVs | 0.563 | 0.486 | 0.303 |
| NC | 0.768 | 0.850 | 0.534 |
| miR-218 inhibitor | 0.778 | - | - |
| NC | 0.694 | - | - |
| miR-218 mimics | 0.045 | - | - |
| miR-218 mimics + NC | 0.039 | - | - |
| miR-218 mimics + TGIF2-ex | 0.855 | - | - |
| ITGBL1-ex | 0.040 | - | - |
| NC | 0.842 | - | - |
| sh-ITGBL1 | 0.93 | - | - |
| sh-NC | 0.844 | - | - |
| The first row is the cell type of fibroblasts; the first column is the name of treatment group.  The values of wound closure area are obtained by the ratio that the migrated cell surface area (24 h) divided by initial surface area (0 h). | | | |

| **Table S4. The collagen gel contraction values in fibroblasts treated with**  **CH/LW-SEVs and related genes** | | | |
| --- | --- | --- | --- |
| Treatment group | CHDFs | LWDFs | HDFs |
| CH-SEVs | 0.456 | 0.559 | 0.356 |
| LW-SEVs | 0.765 | 0.823 | 0.691 |
| NC | 0.995 | 0.964 | 0.975 |
| miR-218 inhibitor | 1.221 | - | - |
| NC | 0.975 | - | - |
| miR-218 mimics | 0.732 | - | - |
| ITGBL1-ex | 0.594 | - | - |
| NC | 0.979 | - | - |
| sh-ITGBL1 | 1.235 | - | - |
| sh-NC | 0.974 | - | - |
| The first row is the cell type of fibroblasts; the first column is the name of treatment group.  The values of collagen gel contraction are obtained by the ratio that the treatment groups divided by the cell contraction inhibitor group. | | | |

| **Table S5. The fold change of total differential miRNAs**  **by RNA**–**seq and qRT**–**PCR** | | |
| --- | --- | --- |
| miRNAs_Name | RNA–seq Log2_FC  (CH-SEVs/LW-SEVs) | qRT–PCR  (CH-SEVs/LW-SEVs) |
| **Upregulation** | | |
| ssc-miR-1306-5p | 2.411 | 0.974 |
| ssc-miR-328 | 2.371 | 1.252 |
| ssc-miR-218 | 2.316 | 3.967 |
| ssc-miR-218-5p | 2.316 | 3.294 |
| ssc-miR-218b | 2.311 | 1.004 |
| ssc-miR-193a-5p | 2.157 | 1.158 |
| ssc-miR-196a | 1.980 | 1.431 |
| ssc-miR-190a | 1.685 | 1.303 |
| ssc-miR-29b | 1.464 | 1.526 |
| ssc-miR-20b | 1.417 | 1.218 |
| ssc-miR-450b-5p | 1.368 | 1.431 |
| ssc-let-7d-3p | 1.324 | 1.038 |
| ssc-miR-423-5p | 1.283 | 0.893 |
| ssc-miR-7134-5p | 1.058 | 0.860 |
| ssc-miR-199a-5p | 1.045 | 1.086 |
| ssc-let-7c | 1.037 | 1.171 |
| ssc-miR-382 | 1.016 | 0.921 |
| **Downregulation** | | |
| ssc-miR-532-5p | -1.106 | 1.444 |
| ssc-miR-146a-5p | -1.401 | 0.546 |
| ssc-miR-141 | -1.994 | 1.520 |
| ssc-miR-182 | -2.252 | 0.820 |
| ssc-miR-429 | -2.317 | 1.171 |
| ssc-miR-200b | -2.322 | 0.104 |
| ssc-miR-335 | -2.985 | 6.857 |
| ssc-miR-205 | -4.198 | 0.183 |
| ssc-miR-34c | -6.257 | 4.685 |
| ssc-miR-31 | -7.347 | 0.197 |

| **Table S6. The quantitative WB data in CHDFs transfected with**  **miR-218 and TGIF2** | | | | | | |
| --- | --- | --- | --- | --- | --- | --- |
| Genes | miR-218 mimics | NC | TGIF2-ex | NC | miR-218 mimics+  TGIF2-ex | miR-218 mimics+NC |
| TGIF2 | 0.025 | 0.107 | 0.196 | 0.069 | 0.091 | 0.005 |
| SMAD2 | 0.073 | 0.019 | 0.007 | 0.016 | 0.015 | 0.100 |
| SMAD3 | 0.039 | 0.015 | 0.003 | 0.015 | 0.016 | 0.030 |
| P-SMAD2/3 | 0.011 | 0.005 | 0.001 | 0.004 | 0.005 | 0.009 |
| TGFB1 | 0.085 | 0.047 | 0.001 | 0.044 | 0.057 | 0.072 |
| Collagen I | 0.296 | 0.176 | 0.023 | 0.164 | 0.148 | 0.201 |
| Fibronectin | 0.045 | 0.013 | 0.008 | 0.010 | 0.012 | 0.048 |
| The first row is the name of treatment group; the first column is gene symbol. | | | | | | |

| **Table S7. The quantitative WB data in CHDFs transfected with miR-218 and TGIF2** | | | | | | |
| --- | --- | --- | --- | --- | --- | --- |
| Genes | miR-218 inhibitor | NC | sh-TGIF2 | sh-NC | miR-218 inhibitor +sh-TGIF2 | miR-218 inhibitor+sh-NC |
| TGIF2 | 0.342 | 0.027 | 0.000 | 0.017 | 0.019 | 0.259 |
| SMAD2 | 0.083 | 0.192 | 0.329 | 0.142 | 0.219 | 0.093 |
| SMAD3 | 0.000 | 0.003 | 0.057 | 0.002 | 0.002 | 0.000 |
| P-SMAD2/3 | 0.000 | 0.003 | 0.030 | 0.002 | 0.002 | 0.001 |
| TGFB1 | 0.036 | 0.099 | 0.124 | 0.083 | 0.089 | 0.026 |
| Collagen I | 0.009 | 0.021 | 0.055 | 0.021 | 0.021 | 0.008 |
| Fibronectin | 0.052 | 0.372 | 0.657 | 0.215 | 0.239 | 0.051 |
| The first row is the name of treatment group; the first column is gene symbol. | | | | | | |

| **Table S8. The quantitative WB data in CHDFs transfected with ITGBL1** | | | | |
| --- | --- | --- | --- | --- |
| Genes | ITGBL1-ex | NC | sh-ITGBL1 | sh-NC |
| ITGBL1 | 0.453 | 0.023 | 0.002 | 0.015 |
| SMAD2 | 0.145 | 0.049 | 0.020 | 0.097 |
| SMAD3 | 0.022 | 0.015 | 0.000 | 0.016 |
| P-SMAD2/3 | 0.024 | 0.011 | 0.001 | 0.008 |
| TGFB1 | 0.066 | 0.030 | 0.006 | 0.019 |
| Collagen I | 0.198 | 0.087 | 0.006 | 0.064 |
| Fibronectin | 0.183 | 0.104 | 0.068 |  |
| The first row is the name of treatment group; the first column is gene symbol. | | | | |
