# Supplementary material for: Small extracellular vesicles derived from dermal fibroblasts promote fibroblast activity and skin development through carrying miR-218 and ITGBL1
Source: J Nanobiotechnology. 2022 Jun 22;20:296. doi: 10.1186/s12951-022-01499-2 (PMC9215004; doi:10.1186/s12951-022-01499-2)
Supplement: Supplementary file 4 — Additional file 4. The detailed materials and methods are provided in supplementary materials and methods and include as follows: TEM, NTA, flow cytometry, RPLC-MS/MS, validation of genes by qRT–PCR, SEVs internalization, cell counting kit-8 assay, 5-Ethynyl-2′-deoxyuridine assay, ELISA assay, wound healing assay, cell cycle analysis, western blot analysis, and animal for SEVs labelling. [file 12951_2022_1499_MOESM4_ESM.docx]

**Additional file 4. Supplementary Material and Methods**

**Transmission electron microscopy analysis (TEM)**

10 μL of samples were absorbed and added with saturated aqueous uranyl to the copper net for several minutes at room temperature. Then the imaging results were obtained by transmission electron microscopy (Hitachi, Japan) at 100 kV.

**Nanoparticle tracking analysis (NTA)**

The samples were diluted 100 times with PBS and detect by nanoparticle tracking analysis (Nanosight NS300, Amesbury, UK) using a NTA2.1 Analytical Software to yield final concentration of 10^10^ particles/mL. All results were recorded.

**Flow cytometry**

The 10^10^ particles/ml of SEVs were incubated with magnetic microbeads coated with CD9 or CD63 at 4 °C for 1 h. The labelled SEVs were washed thrice with buffer, and antibody attached SEVs were incubated with their corresponding IgG secondary antibody for 30 min on ice. Finally, SEVs surface markers of CD9 and CD63 were analysed by flow cytometry.

**Protein detected by reversed-phase liquid chromatography-tandem mass spectrometry (RPLC-MS/MS)**

SEVs were extracted by 1 ml of lysis buffer and quantified by BCA assay. The 3 mg of protein was digested using filter-aided sample preparation method. Then, the protein pellets were re-suspended in DTT buffer with ammonium hydrogen carbonate (50 mM) at 56 °C for 40 min, and added 50 mM IAA at RT for 20 min in the dark. The mixture was centrifuged at 12,000 × g for 10 min after adding 50 mM Urea and ABC. Then, trypsin digestion (1:50 protease to protein ratio) and 50 mM ABC was added to each sample following by incubating at 37 °C for 16-18 h. The polypeptide was obtained and implemented analysis by Reversed-Phase Liquid Chromatography-Tandem Mass Spectrometry (RPLC-MS/MS). In briefly, the flow rate was 300 nL/min to proceed on Acclaim PepMap C18. The mobile phases consisted of 0.1% acetic acid, 80% acetonitrile and linear gradient of 3–7% B in 5 min, 7–20% B in 60 min, 20–32% B in 90 min, and 90% B for 120 min. Finally, the mass spectrometry data was obtained using Orbitrap Fusion Lumos Mass Spectrometer (Thermo Scientific).

**Validation of genes by qRT**–**PCR**

Total SEV RNA and cell RNA was extracted using TRIzol reagent according to the manufacturer’s protocols (Invitrogen, CA, USA). Reverse transcription of mRNA and miRNA was performed with a commercial kit according to the manufacturer’s protocol (Takara, Shiga, Japan). qRT–PCR was used to identify the expression levels of selected genes. To amplify specific fragments of selected genes, specific primers were designed with NCBI Primer-BLAST (Table 1). ACTB was used as control to normalize the expression level of these targeted genes. All samples were amplified in triplicate, and the mean and standard error values were calculated.

| **Table 1. The sequences of the primers used for qRT**–**PCR** | | |
| --- | --- | --- |
| Gene | Forward Primer (5ʹ﹘3ʹ) | Reverse Primer (5ʹ﹘3ʹ) |
| miR-1306-5p | CCACCTCCCCTGCAAACGTCCA | Universal qPCR Primer included in kit (miRNA universal downstream primer, TaKaRa) |
| miR-328 | CTGGCCCTCTCTGCCCTTCCGT |  |
| miR-218 | TTGTGCTTGATCTAACCATGT |  |
| miR-218-5p | TTGTGCTTGATCTAACCATGT |  |
| miR-218b | TTGTGCTTGATCTAACCATGTG |  |
| miR-193a-5p | TGGGTCTTTGCGGGCGAGATGA |  |
| miR-196a | TAGGTAGTTTCATGTTGTTGGG |  |
| miR-190a | TGATATGTTTGATATATTAGG |  |
| miR-29b | TAGCACCATTTGAAATCAGTGTT |  |
| miR-20b | CAAAGTGCTCACAGTGCAGGTAG |  |
| miR-450b-5p | TTTTGCAATATGTTCCTGAATA |  |
| let-7d-3p | CTATACGACCTGCTGCCTTTCT |  |
| miR-423-5p | TGAGGGGCAGAGAGCGAGACTTT |  |
| miR-7134-5p | ATGTCCGCGGGTTCCCTATCC |  |
| miR-199a-5p | CCCAGTGTTCAGACTACCTGTTC |  |
| let-7c | TGAGGTAGTAGGTTGTATGGTT |  |
| miR-382 | AAGTTGTTCGTGGTGGATTCG |  |
| miR-532-5p | CATGCCTTGAGTGTAGGACCGT |  |
| miR-146a-5p | TGAGAACTGAATTCCATGGGTT |  |
| miR-141 | TAACACTGTCTGGTAAAGATG |  |
| miR-182 | TTTGGCAATGGTAGAACTCACACT |  |
| miR-429 | TAATACTGTCTGGTAATGCCGT |  |
| miR-200b | TAATACTGCCTGGTAATGATGAC |  |
| miR-335 | TCAAGAGCAATAACGAAAAATG |  |
| miR-205 | TCCTTCATTCCACCGGAGTCTG |  |
| miR-34c | AGGCAGTGTAGTTAGCTGATTGC |  |
| miR-31 | AGGCAAGATGCTGGCATAGCTG |  |
| H-COL1A1 | CTCTGGTGAACCTGGCAAAC | AGCCACGGTGACCCTTTATG |
| P-COL1A1 | CTGGAGAAGAAGGAAAGCGAG | GAAACCACGGCTACCAGGTC |
| TGIF2 | GGCAAAGACCCCAACCAGTA | GTACAGACACAGCCAGCACT |
| TGFB1 | TTACAACAGTACCCGCGACC | CCGCTTTCCAGCATTAGCAC |
| TGFB2 | TTCTTCCCCTCGGAAAATGCC | TGTTATATAAGCTGAGAACCCTGC |
| P-ACTB | TGGATGACGATATTGCTGCG | GGGTCAGGATGCCTCTCTTG |
| H-ACTB | GGCTGTGCTATCCCTGTACG | CTTGATCTTCATTGTGCTGGGTG |

**SEVs internalization**

Cells (10^5^) of CHDFs were treated with 10 μg of SEVs (100 ﻿μg/mL) labelled by PKH67 in DMEM medium in confocal dish. As negative control (NC), 100 ﻿μL of supematant free SEVs-labelled was used in the experiment. After incubation for 24 h, the cells were washed with PBS and fixed in a solution of 10% neutral buffered formalin for 15 min. Then, cell nucleus were stained with DAPI dye for 30 min and were imaged using Leica laser confocal scanning.

**Cell Counting Kit-8 assay (CCK-8)**

The ~ 4×10^4^ cells proliferation was detected using Cell Counting Kit 8 (Beyotime, Shanghai, China). Fibroblasts were seeded in 96-well plates and cultured for 12 h. Then CHDFs/LWDFs/HDFs were treated with DMEM with 4 μg of CH/LW-SEVs (10^10^ particles of /ml). As negative control (NC), 40 ﻿μL of PBS was added into DMEM. After 24 h, 10 μL of CCK-8 was added into the treated fibroblasts for 2 h at 37 °C with 5% CO_2_ and assessed the results at 0 h, 24 h, 48 h and 72 h using Varioskan LUX instrument (Thermo Scientific).

**5-Ethynyl-2′-deoxyuridine assay (EdU)**

The ~ 1×10^4^ cells proliferation was evaluated using a 5-ethynyl-2′-deoxyuridine assay (Beyotime, Shanghai, China). For the EdU analysis, fibroblasts were incubated with 10 μM of EdU for 2 h at 37 °C with 5% CO_2_ after treament with DMEM with 4 μg of CH/LW-SEVs (10^10^ particles of ml) and 40 ﻿μL of PBS as NC in CHDFs/LWDFs/HDFs, miR-218 mimics/inhibitor, ITGBL1/TGIF2 plasmid vectors, ITGBL1/TGIF2 interfering, or NC for 24 h. EdU staining was performed according to the manufacturer’s protocol. Images were captured using an Olympus IX53 microscope (Olympus, Tokyo, Japan).

**ELISA assay**

The skin dermis fibroblasts were treated basing on different experimental conditions, which were divided into CH/LW-SEVs, NC, miR-218 mimics/inhibitor, ITGBL1/TGIF2 plasmid vectors, or ITGBL1/TGIF2 interfering. Moreover, the mouse skin was dissociated by 300 μL of lysis to obtain skin protein re-supernatant. Then the supernatants of all groups were collected. According to the Collagen I ELISA Kit instructions (EIAab, Wuhan, China), we constructed a standard curve for each set of experimental samples. Detection A and Detection Reagent B solutions were incubated for 1 h and 45 min at 37 °C, respectively. Then, the substrate was incubated in dark for 20 min at 37 °C, and we finally estimated the result at 450 nm using Varioskan LUX instrument (Thermo Scientific) after adding Stop Solution.

**Wound healing assay**

The ~7×10^6^ Cells were seeded in 6-well dishes to create a confluent monolayer. The cell monolayer was scraped with a p200 pipette tip in a straight line to create a "scratch" after treated with 10 μg of SEVs (100 ﻿μg/mL) from CHDFs and LWDFs in CHDFs/LWDFs/HDFs, miR-218 mimics/inhibitor, ITGBL1/TGIF2 plasmid vectors, ITGBL1/TGIF2 interfering, or NC. The wound area was measured between 0 h and 24 h by Image J. The wound closure area is obtained by the ratio that the migrated cell surface area (24 h) values divided by initial surface area (0 h).

**Cell Cycle analysis**

The ~7×10^6^ cells were treated with 10 μg of SEVs (100 ﻿μg/mL) from CHDFs and LWDFs in CHDFs/LWDFs/HDFs, miR-218 mimics/inhibitor, ITGBL1/TGIF2 plasmid vectors, ITGBL1/TGIF2 interfering, or NC. After 24 h, the cells were digested from 6-well dishes, washed and fixed in 75% alcohol and incubated with PI/Rnase staining buffer at 4 ºC for 30 min. The cell resuspension solution was detected by flow cytometry (Beckman Coulter, USA) and the data of ratio for different stages of the cell cycle was analyzed by Modfit.

**Western blot analysis**

SEVs protein and ~7×10^6^ cell protein were extracted according to the protocol of the Tissue or Cell Total Protein Extraction Kit (Sangon, Shanghai, China). Then, the protein concentration was measured by the BCA Assay Kit instructions (Coolaber, Beijing, China). The total proteins were diluted with 5x protein SDS PAGE loading. Then, the protein was separated by 10% SDS-PAGE at 90 V and transferred to PVDF membranes for 2 h. We used the related protein antibodies to incubate the membranes at 4 °C overnight (Table 2). The secondary used antibody was HRP-conjugated goat anti-rabbit IgG (1:6000), which was incubated with shaking for 2 h at room temperature. Finally, the images were visualized with ECL solution (Sangon, Shanghai, China) using the Touch Imager XLi (e-BLOT, China). According to the gray scanning of bands (bands intensities), the proteins expression levels of each band were quantified by e-BLOT software, which normalized to the levels of GAPDH (as loading control) in the same samples.

| **Table 2. These list of related protein antibodies** | | |
| --- | --- | --- |
| Protein name | Manufacturer's name and location | Source |
| CD9 | Sangon, Shanghai, China (1:1000) | rabbit polyclonal  antibody |
| CD63 | Sangon, Shanghai, China (1:1000) |  |
| CD81 | Affinity, OH, USA (1:1000) |  |
| ITGBL1 | Affinity, OH, USA (1:1000) |  |
| TGIF2 | Affinity, OH, USA (1:1000) |  |
| Collagen I | Sangon, Shanghai, China (1:1000) |  |
| Fibronectin | Affinity, OH, USA (1:1000) |  |
| SMAD2 | Affinity, OH, USA (1:1000) |  |
| SMAD3 | Affinity, OH, USA (1:1000) |  |
| p-SMAD2/3 | Affinity, OH, USA (1:1000) |  |
| TGFβ1 | Affinity, OH, USA (1:1000) |  |
| GAPDH | Sangon, Shanghai, China (1:6000) |  |

**Animal for SEVs labeling**

The 100 μL of SEVs (150~200 ﻿μg/mL) labelled with DiR dye were injected by subcutaneous into the buttocks of mouse mice. The supematant of free SEVs-labelled was used as a negative control. After injection 1 day, the nude mice were imaged by IVIS imaging. Then, the mice were slaughtered and the mouse skin tissues were frozed to immunofluorescent staining; in addition, cell nucleus were stained with DAPI dye.
